# Epidemiological Investigation of Type 2 Diabetes and Alzheimer’s Disease in a Pakistani Population †

**DOI:** 10.3390/ijerph15081582

**Published:** 2018-07-26

**Authors:** Zarish Noreen, Jessica DeJesus, Attya Bhatti, Christopher A. Loffredo, Peter John, Jahangir S. Khan, Gail Nunlee-Bland, Somiranjan Ghosh

**Affiliations:** 1 Department of Biology, Howard University, Washington, DC 20059, USA; zarish.noreen@gmail.com; 2 Department of Healthcare Biotechnology, National University of Sciences and Technology (NUST), Islamabad 44000, Pakistan; attyabhatti@gmail.com (A.B.); pjohn72@hotmail.com (P.J.); 3 Departments of Oncology and of Biostatistics, Georgetown University, Washington, DC 20057, USA; jd1764@georgetown.edu (J.D.); cal9@georgetown.edu (C.A.L.); 4 Department of Surgery, Rawalpindi Medical College, Rawalpindi, Punjab 46000, Pakistan; jskdr@hotmail.com; 5 Departments of Pediatrics and Child Health, College of Medicine, Howard University, Washington, DC 20059, USA; gnunlee-bland@howard.edu

**Keywords:** type 2 diabetes, Alzheimer’s disease, Pakistan, epidemiology

## Abstract

The epidemic of type 2 diabetes mellitus (T2DM) and the possibility of it contributing to the risk of Alzheimer’s disease (AD) have become important health concerns worldwide and in Pakistan, where the co-occurrence of T2DM and AD is becoming more frequent. To gain insights on this phenomenon, a cross-sectional study was initiated. We recruited and interviewed 820 research participants from four cities in Pakistan: 250 controls, 450 T2DM, 100 AD, and 20 with both diseases. Significant differences between groups were observed for age (*p* < 0.0001), urban vs. rural locality (*p* = 0.0472) and residing near industrial areas. The average HbA1c (%) level was 10.68 ± 2.34 in the T2DM group, and females had a lower level than males (*p* = 0.003). In the AD group, significant relationships existed between education and family history. Overall, the results suggest that T2DM and AD were associated with both socio-demographic and environmental factors in Pakistani participants. Detailed molecular investigations are underway in our laboratory to decipher the differential genetic pathways of the two diseases to address their increasing prevalence in this developing nation.

## 1. Introduction

Type 2 diabetes mellitus (T2DM)—the most common form of diabetes mellitus (DM)—is an extremely chronic, heterogeneous, and multifactorial metabolic disorder characterized by hyperglycemia, insulin resistance, and insulin insufficiency [1]. T2DM is one of the most predominant and serious health problems worldwide and accounts for 90% of diabetes cases [2]. It is more affected by multifactorial environmental and genetic factors [3]. Its prevalence is dramatically increasing in developing countries as compared to developed countries. It has been predicted that in the next 20 years, the prevalence of T2DM will continue to rise with more than 70% of cases in developing countries [4]. According to statistical data, six countries of developing and lower middle income (India, China, Brazil, Indonesia, Bangladesh, and Pakistan) are in the top tier of T2DM prevalence, and Pakistan is ranked sixth worldwide in 2000, where the diabetic prevalence was 5.2 million in 2000, and is estimated to be 13.9 in 2030 [5]. 

Pakistan has suffered epidemiological consequences of economic development. The growing urbanization in Pakistan has led to sedentary lifestyle, high-calorie food intake, lack of exercise, and more stressful living conditions, all of which may be related to the risk of developing T2DM. Although genetic factors are well recognized for contributing to T2DM, recent research has emphasized the importance of environmental factors like dietary changes, age, lack of exercise, obesity, and inactive lifestyle, and their differential impacts in men and women [6,7].

T2DM affects different organs including the brain. One of the major complications of T2DM is increased risk of developing Alzheimer’s disease (AD) [8]. It is one of the major health issues of 21st century with both genetic and environmental factors involved in its pathogenesis [9]. According to recent reports, currently almost 35 million people worldwide are affected with AD including 5.4 million Americans, two-thirds of whom are women. This number is estimated to be double in the next 20 years [10]. Research has shown higher prevalence of AD in women due to the longer life expectancy of females, on average [11]. 

T2DM and AD share certain common cellular and molecular mechanisms in pathogenesis. T2DM is linked with impairment of the cognitive function, and these diabetic patients have twice the risk of developing AD [12,13]. Studies showed that there are several regions in the brain which are affected in the T2DM individual resulting in the poorer cognitive presentation [14]. Individuals with T2DM have increased brain atrophy linked with certain cognitive impairments [15,16]. Patients with T2DM as compared to the controls have less performance in terms of cognitive ability, attention, recalling memory, information processing speed, and other functioning. These studies showed that cognitive function and mental health and flexibility are affected in T2DM cases [17,18].

Currently, there is a rapid growth in the literature pointing towards insulin deficiency and insulin resistance as mediators of AD-type neurodegeneration, but this surge of new information is riddled with conflicting and unresolved concepts regarding the potential contributions of T2DM, metabolic syndrome, and obesity to AD pathogenesis [19]. The term “type 3 diabetes” (T3DM) may accurately reflect the fact that AD represents a form of diabetes that selectively involves the brain and has molecular and biochemical features that overlap with both type 1 diabetes mellitus and T2DM [20]. Recent studies showed that insulin genes are also expressed in human brain and the deficiency of insulin, insulin growth factor 1 (IGF-1), insulin growth factor 2 (IGF-2), and their conforming receptors could be interconnected in the development of neurodegeneration even in the absence of T2DM [21]. The insulin growth factors (IGFs) play a significant role in the pancreatic islets functioning [22,23].

T2DM and AD both have higher prevalence in the aging population. Due to common pathogenic factors underpinning both diseases, T2DM has emerged as a significant risk factor for prognostic development of AD. In the present report, we present descriptive information about the study population—highlighting associations between T2DM, AD, and socio-demographic and environmental factors—as a prelude to future research on the interplay of these factors with genetics. 

## 2. Materials and Methods

### 2.1. Study Population 

**Figure 1:**
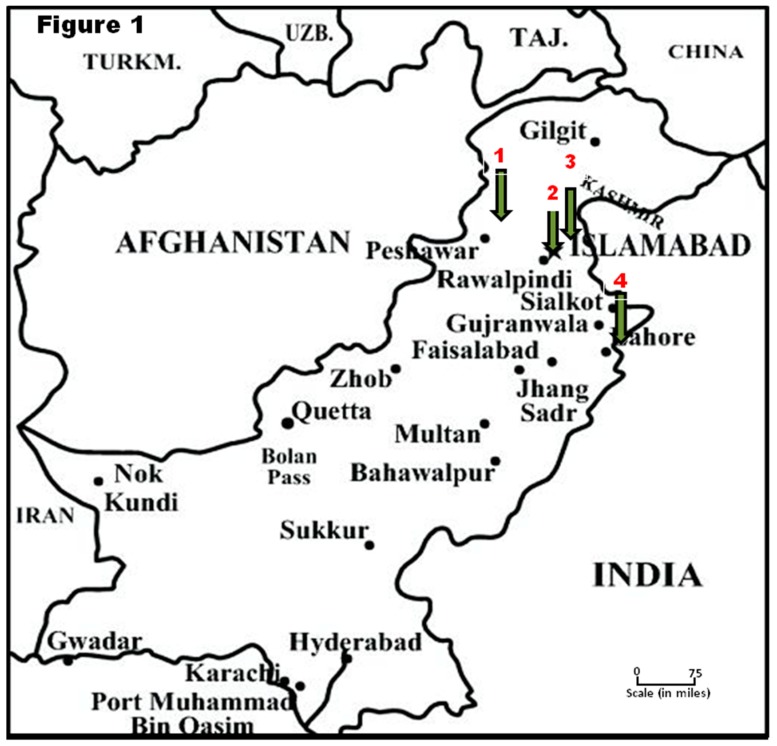
The study area where from the human volunteers (research participants) were recruited under the present investigation with type of areas in parenthesis: (**1**) Abbottabad (Rural); (**2**) Rawalpindi (Urban); (**3**) Islamabad (Urban); and (**4**) Lahore (Industrial).

Subjects for this study were a convenience sample, recruited from the out-patient departments of four different regional hospitals of Pakistan, i.e., Islamabad, Rawalpindi, Lahore, and Abbottabad (Figure 1). The study was undertaken with the prior approval by the Howard University Institutional Review Board (IRB-17-MED-43), and the informed consent was obtained from volunteers as per the approval of the Institutional Review Board (IRB) of Atta-ur-Rahman School of Applied Biosciences (ASAB), National University of Sciences and Technology (NUST) (28/IRB; dated. 20 April 2016). A total of 820 research participants were recruited. The recruitment was carried out during February 2016 and May 2017. Among the subjects were 250 controls (lacking T2DM and AD), 450 with T2DM, 100 with AD, and 20 with both T2DM and AD. A routine questionnaire was administered to collect information about education, family history of T2DM, place of residence (urban vs. rural), and proximity of the residence to industrial pollution. For AD patients, family members helped to answer the questions. The analysis reported below was performed free of personal identifiers.

### 2.2. Hematological and Clinical Investigation 

Clinical diagnosis was performed by physicians (endocrinologists for T2DM) and by the neurologists (for AD) in the respective hospitals. The fasting blood glucose (mg/dL) was measured by blood glucose glucometer, and HbA1c % test was performed by automated machine (7180 Clinical Analyzer, Hitachi, Tokyo, Japan) in diagnostic laboratories to diagnose T2DM—i.e., participants with HbA1C 6.7% or higher were considered as diabetic, according to guideline of American Diabetic Association [24]. Medical interviews, neurological examinations, and magnetic resonance imaging (MRI) were performed to diagnose AD patients in which symptoms of Alzheimer’s, such as memory loss, word-finding difficulties, and visual/spatial problems, are significant enough to impair a person’s ability to function independently, along with interviews with the person as well as a family member, friend, or caregiver about changes in the person’s thinking skills, as per the Alzheimer’s Disease Diagnostic Guidelines of National Institute of Aging (NIA/NIH: https://www.nia.nih.gov/health/alzheimers-disease-diagnostic-guidelines) as well as recommendations from the National Institute on Aging-Alzheimer’s Association workgroups on diagnostic guidelines for Alzheimer’s disease [25].

### 2.3. Statistical Analysis 

The continuous variables of age, plasma glucose (mg/dL), and HbA1c (%) level were not normally distributed, and hence we used non-parametric methods for their analysis. The age variable was categorized into three groups: age group 1 includes ages less than or equal to 55, age group 2 includes ages greater than or equal to 56 and less than 70, and age group 3 includes ages greater than or equal to 70. The variables gender, locality (urban vs. rural), education (formal education vs. no formal education), living near an industrial area (yes/no), and family history (yes/no for AD and separately for T2DM) were analyzed as categorical variables. Associations among the categorical variables were determined using chi-squared tests. To test differences between HbA1c (%) level and categorical variables, Wilcoxon two-sample tests, Kruskal–Wallis, and median one-way analysis tests were used. Quantile regression was used to further examine relationships between HBA1c and the categorical variables. The values *p* < 0.05 were considered statistically significant. Statistical analysis was done using SAS 9.4 statistical software (SAS Institute Inc., Cary, NC, USA).

## 3. Results

### 3.1. Type 2 Diabetes 

The T2DM group was comprised of 56% females and 42% males. The highest and the lowest HBA1c (%) level recorded for T2DM patients were 13.8 and 5.5 respectively. The highest and the lowest plasma glucose recorded were 560 mg/dL and 102 mg/dL, respectively. The average HbA1c (%) level in T2DM participants was 10.86, which was 2.2 times higher as compared to AD and control subjects, but very similar to the level observed for subjects with T2DM + AD. Compared to the other three study groups, those with T2DM were less likely to report having no formal education and living in urban areas. A slight majority of the T2DM participants reported having no family history of diabetes (Table 1).

After performing chi-squared tests to determine associations between groups (Control, AD, T2DM, and both T2DM and AD) and sociodemographic variables, significant associations were found with age groups (*p* < 0.0001) and localities (*p* = 0.0472). Participants in age group 1 (ages less than or equal to 55) represented the highest percent in the T2DM group, while participants in age group 3 (ages greater than or equal to 70) had the highest percent in the AD and the both T2DM and AD group. The majority of participants lived in urban localities T2DM group as well, and in the group with both T2DM and AD (Table 2).

**Figure 2:**
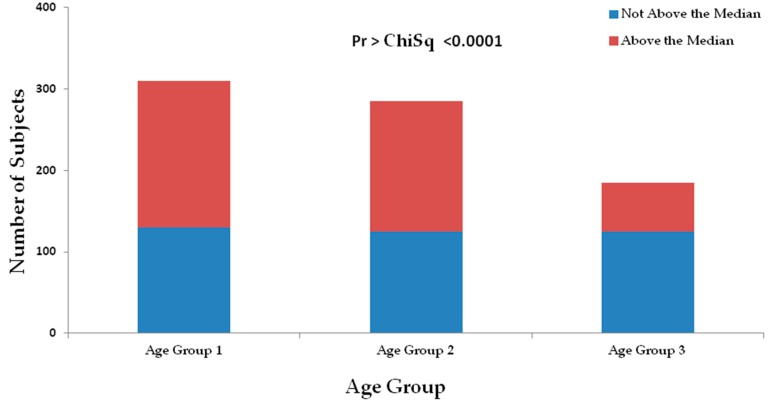
Displaying the frequency counts of research participants above and below the median HBA1c Level percent of 7.1% for each age group. Age was categorized by the following groups: Age group 1 (less than or equal to 55 years old), age group 2 (greater than or equal to 56 years and less than 70 years old), age group 3 (greater than or equal to 70 years old).

A significant relationship was found between HbA1c (%) level and age group (Kruskal–Wallis test *p* < 0.0001, median one-way analysis *p* < 0.0001), with the youngest age group having the most values above the sample HbA1c (%) level median of 7.1 (Figure 2).

### 3.2. Alzheimer’s Disease 

The AD participants enrolled in this study comprised more males (55%) than females (45%). in the studied AD participants 57% were living in urban areas compared to rural areas (43%) and 46% had formal education. Furthermore, only 31% of AD participants had family history of disease and the majority (70%) were not living in or close to industrial areas (Table 1).

Within the AD group, significant relationships existed between HbA1c (%) level and education, and HbA1c (%) level and family history. The median of HBA1c % level for AD group participants who had no formal education was 0.8% higher than educated participants (*p* = 0.0003). The median of HbA1c (%) level for AD group participants who did not have a family history of AD was 0.7% higher than participants with a family history of AD (*p* = 0.0042). 

### 3.3. T3DM

A small group with both T2DM and AD (i.e., T3DM, *N* = 20) were also enrolled in this study. The HbA1c (%) level in these participants was lower as compared to the group with only T2DM. The majority of them did not have any family history of T2DM and AD, and more were from rural areas as compared to urban areas (Table 1). The small size of this group precluded further formal statistical testing.

## 4. Discussion

The present descriptive study was designed to investigate potential risk factors and shared links between T2DM and AD in Pakistani population. Out of the total research participants who were recruited from four cities of Pakistan, 450 were T2DM patients, including 57.5% females and 42.5% male. The percentage of T2DM females was higher as compared to males, which is consistent with previous studies [5,6,26]. The females in Pakistan have a lower education level, less aware of their disease, higher obesity rate and less physically active as compared to males which may account for their differential risk of developing diabetes [27]. 

Our data suggest that the larger percentage of T2DM participants belong to the youngest age group we studied (≤55 years). Numerous studies have shown that T2DM is more common in middle age, and age is one of the major risk factors [28,29]. Previous studies also revealed that T2DM in an Asian population is more common in middle age, as compared to western populations [30]. This earlier onset of T2DM is a significant factor that influences the future burden of this disease, with increased morbidity and mortality rates. 

The majority of our patients with T2DM lived in urban areas as compared to rural areas. This finding is consistent with previous studies, which have reported that T2DM prevalence in urban areas has been dramatically increased in many countries [31,32]. An intermediate rate (30%) of urbanization has been observed in Pakistan, China, Thailand, and India, and studies have predicted that further increases in urbanization will occur in the majority of Asian countries, including Pakistan [33]. The cause of this abrupt rise in T2DM can be ascribed to a number of factors, the most important being urbanization, rural to urban migration, modernization, industrialization, readily available fast foods, and western lifestyle.

Glycemic control is the significant therapeutic objective to control T2DM-related complications. The HbA1c test is used to monitor long term glycemic control over the disease management [34]. The results from our study showed alarming average HbA1c (%) level of 10.86 in T2DM participants, which is 2.22 times higher as compared T3DM and control subjects. These results are in agreement with the previously conducted studies in Pakistan where 81.3% of Karachi and 46.7% of Rawalpindi populations had values exceeding the optimal range and >8.5% was observed in T2DM patients with ≤55 years of age [35,36].

Our study also observed that a participant’s education level was not associated with T2DM, and half of those participants had no formal education. This is largely consistent with a previous study conducted in Peshawar that reported that almost 58% of T2DM patients had no formal education, lacked adequate knowledge of the disease, and had an average HbA1c level of 9.98% [37].

Our findings suggest that 52% of the T2DM patients do not report family history of disease. These findings are divergent to previous studies [38]. People with family history of T2DM have six times higher risk of developing T2DM as compared to people with no family history [39,40].

The increased incidence of these two diseases has become major public health issue in Pakistan. AD and other dementias disproportionately affect women. The Lancet Neurology Commission [41] affirms that, for most regions of the world, the occurrence of AD and other dementias is higher in women than in men, particularly in the most elderly [42]. The main risk factor for AD is age, but gender is also very important. The proportion of persons suffering AD is always higher in women than in men. The increase in life expectancy in western countries, particularly the US and Europe, has been associated with a parallel increase in age-related diseases [43]. The onset of a hypometabolic phenotype in female brains could serve as an important mechanistic rationale for female susceptibility to AD [44]. Among our study participants, 55% of males and 45% of females were reported having AD. Vascular dementia is often misdiagnosed as AD, and vascular dementia are more common in males than females, because of atherosclerosis, the major cause vascular dementia, is more common in men than women. Hence, these differences may have arisen in the reported cases due to misdiagnosis or selection bias. 

In our study population, the majority of AD patients (57%) were from urban areas. This is in contrary with the previous studies which showed that AD is more common in rural areas as compared to urban areas [45,46]. We note with caution, however, that our observation may reflect selection bias and should not be generalized.

The possibility of developing AD increases with the increase in age and this risk doubles every five years after the age of 65. The abnormal accumulation of insoluble protein masses, deposition of toxic proteins, and problems in specific conserved mechanisms like DNA repair, cellular maintenance pathways, and metabolic homeostatic are associated with aging resulting in progressive death of neurons and loss of brain structures, making advanced age the important risk factor for AD development [47,48]. The mean age in our study was found to be 79.4. The results of earlier studies confirmed the incidence of AD increases in older age and are more common after 65 years of age [49,50]. Besides other risk factors, apolipoprotein E (*ApoE*) gene polymorphism has been implicated in predisposition to diabetes and dementia in old population, but the results from the different studies were inconclusive. However, in Pakistani population *APO E4* alleles and ATP7B variant alleles are known genetic factors increasing the risk of AD [51], which is also corroborated with a the results of a large prospective cohort study confirmed case–control reports that women who are positive for the ε4 allele of the apolipoprotein E gene (*APOE ε4*) are at greater risk of developing AD [52].

Level of education is a well-established risk factor for AD but its relation to cognitive decline, the prime clinical expression of the disease, is unsure. Consistent with this idea, several studies have reported an association of higher educational attainment with reduced cognitive decline [53]. Studies have also reported that the people with higher levels of education tend to have reduced risk of developing AD in old age, and also found associations of higher education with reduced cognitive impairments [54,55]. A higher level of education may help hold off severe cognitive decline in old age [56]. A significant association between the risk of AD and lower education level was also observed that corroborated in present findings [57]. Other findings also indicated significant association between T2DM group, age, education, and area of locality [58,59,60], that matched our observation. These demographic parameters directly or indirectly are associated with T2DM and AD. Thus lifestyle modification, awareness, and patient education might play role in disease management and reducing its incidence. 

Our study had some strengths, including the standardized questionnaire and medical examinations that were applied consistently across the research centers However, the results are limited by small sample size of the T3DM group, and lack of some potentially important variables like BMI, medication usage, and co-morbidities such as hypertension. The family history status for T2DM and AD both were reported by the research participants and we did not inspect the patient relative’s medical records ourselves. The other major limitation is the convenience sampling from selected regions, pointing out the need for future randomized sample collection that would be more broadly representative of the Pakistani population.

## 5. Conclusions

Our study confirms that T2DM and AD are associated with certain socio-demographic factors, e.g., area of residence, lack of education, and age, which may have collectively influenced the risks of developing T2DM and AD in this population. In-depth molecular investigations are underway using biospecimens collected from the participants to decipher genetic pathways of these two diseases to address the increasing prevalence of these two chronic diseases in this developing nation.

## Figures and Tables

**Table 1:** Demographic and clinical data of the study population.

Variables	Control	T2DM	AD	T2DM + AD
	(*n =* 250)	(*n =* 450)	(*n =* 100)	(*n =* 20)
Age (years):				
Mean ± STD	58.65 ± 12.2	56.85 ± 12.06	79.40 ± 7.93	79.30 ± 7.37
Sex (*N*, %)				
Male	114 (46)	170 (42)	55 (55)	11 (55)
Female	136 (54)	230 (58)	45 (45)	9 (45)
Locality (*N*, %)				
Rural	97(39)	158 (35)	43 (43)	9 (45)
Urban	153 (61)	292 (65)	57 (57)	11 (55)
Education (*N*, %)				
Formal Education	101 (41)	224 (50)	46 (46)	8(40)
No Formal Education	149 (59)	226 (50)	54(54)	12(60.0)
Close to Industry (%)				
Yes	72 (29)	180 (40)	30 (30)	8(40)
No	178 (71)	270 (60)	70 (70)	12 (60)
Family History (*N*, %)				
Yes	NC	217 (48)	31(31)	7(35)
No	NC	233 (52)	69 (69)	13(65)
HbA1c (%)	4.89 ± 0.73	10.68 ± 2.34	5.35 ± 0.76	10.57 ± 1.69
Glucose (mg/dL)	101.38 ± 13.13	336.99 ± 102.78	109.00 ± 10.01	267.50 ± 71.04

HbA1c—hemoglobin A1c; NC: Data not captured; Sex (M/F) information was missing in 50 subjects.

**Table 2:** Chi-squared test: group vs. age and locality.

Group	Age Group-1	Age Group-2	Age Group-3	Locality	Locality	Total
	(≤55 Year)	(≥56 < 70 Year)	(≥70 Year)	(Rural)	(Urban)	
Frequency Control	111	102	37	97	153	250
Row Percent	44.4 *	40.8		14.8	38.8	61.2 **
Frequency AD	0	16	84	54	46	100
Row Percent	0	16	84 *	54 **	46	
Frequency T2DM	200	150	50	158	242	450
Row Percent	50 *	37.5		12.5	39.5	60.5 **
Frequency T2DM	0	3	17	9	11	20
Row Percent + AD	0	15		85 *	45	55 **
Frequency TOTAL	311	271	188	318	452	820

* Group vs. age group chi-squared test *p*-value: <0.0001; ** group vs. locality chi-squared test *p*-value: 0.047.
